# Acute Effects of Different Stretching Techniques on the Number of Repetitions in A Single Lower Body Resistance Training Session

**DOI:** 10.1515/hukin-2015-0018

**Published:** 2015-04-07

**Authors:** Marcos A. Sá, Gabriel R. Neto, Pablo B. Costa, Thiago M. Gomes, Cláudio M. Bentes, Amanda F. Brown, Jefferson S. Novaes

**Affiliations:** 1 Federal University of Rio de Janeiro. Postgraduate Program in Physical Education Stricto Sensu/ UFRJ. Rio de Janeiro, Brazil.; 2 Department of Physical Education, Associate Graduate Program in Physical Education UPE / UFPB, João Pessoa, Paraíba -Brazil.; 3 California State University, Fullerton. Department of Kinesiology. Fullerton, California.; 4 University Estacio de Sá, Rio de Janeiro, Rio de Janeiro - Brazil.

**Keywords:** warm-up, ballistic stretching, passive stretching, muscular strength, lower limbs

## Abstract

This study aimed to investigate the acute effects of passive static and ballistic stretching on maximal repetition performance during a resistance training session (RTS). Nine male subjects underwent three experimental conditions: ballistic stretching (BS); passive static stretching (PSS); and a specific warm-up (SW). The RTS was composed of three sets of 12RM for the following exercises: leg press 45 (LP), leg extension (LE), leg curl (LC), and plantar flexors (PF). Performance of six sessions was assessed 48 hours apart. The first visit consisted of a familiarization session including stretching methods and exercises used in the RTS. On the second and third visit, a strength test and retest were performed. During the fourth to the sixth visit, the volunteers randomly performed the following protocols: BS+RTS; PSS+RTS; or SW+RTS. For the sum of the RM number of each three-set exercise, significant differences were found between PSS vs. SW for the LP (p = 0.001); LE (p = 0.005); MF (p = 0.001); and PF (p = 0.038). For the comparison between the methods of stretching PSS vs. BS, significant differences were found only for the FP (p = 0.019). When analyzing the method of stretching BS vs. SW, significant differences were found for the LP (p = 0.014) and MF (p = 0.002). For the total sum of the RM number of three sets of the four exercises that composed the RTS, significant differences were observed (p < 0.05) in the following comparisons: PPS vs. SW (p = 0.001), PPS vs. BS (p = 0.008), and BS vs. SW (p = 0.002). Accordingly, the methods of passive static and ballistic stretching should not be recommended before a RTS.

## Introduction

According to the American College of Sports Medicine (2011), several components comprise physical fitness, including body composition, cardiorespiratory, neuromotor, and muscular fitness, which influence positively the quality of life. The strength and flexibility components of muscular and neuromotor fitness have sparked the interest of many researchers and have been the subject of numerous scientific studies (Sá et al., 2013; Barroso et al., 2012; Gomes et al., 2011). Numerous authors presented results from investigations related to the acute effects of different stretching methods on subsequent strength performance (Rubini et al., 2007; Barroso et al., 2012). These studies compared the acute effects of static stretching on power performance (Yamaguchi et al., 2006), peak torque (Cramer et al., 2004; 2006; 2007), muscle damage (Chen et al., 2014), muscular endurance (Franco et al., 2008), maximum voluntary contraction (Ogura et al., 2007), and maximum 1RM strength (Beedle et al., 2008; Bacurau et al., 2009; Winchester et al., 2009). Acute effects of dynamic stretching on strength (Costa et al., 2014), muscle power (Manoel et al., 2008), peak torque (Ayala et al., 2013; Costa et al., 2014; Sekir et al., 2010), muscle imbalance (Costa et al., 2014), and muscle activation (Costa et al., 2014; Sekir et al., 2010) were also investigated. However, no studies have examined the acute effects of static and dynamic stretching on performance of an entire resistance training session.

Winchester et al. (2009) investigated whether the number of sets of static stretching performed would decrease the maximum strength on a knee flexion exercise 1RM test. The authors reported that one set of static stretching was sufficient to reduce the levels of strength by 5.4%, while six sets promoted further reduction of 12.4%. Similarly, Gomes et al. (2011) examined the effects of static and proprioceptive neuromuscular facilitation (PNF) stretching on the maximum number of repetitions at 40, 60, and 80% intensity of 1RM in the knee extension exercise. The authors found no significant differences in strength performance after static stretching when compared to the no-stretching condition. In contrast, Bacurau et al. (2009) reported reductions in strength in the leg press exercise for passive static stretching when compared to ballistic stretching (2.2%) and the control group (13.4%). However, the authors did not report significant differences between ballistic stretching and control groups. Likewise, Barroso et al. (2012) compared the acute effects of static, ballistic, and PNF stretching on the number of repetitions for the leg press. The authors reported that all stretching protocols significantly reduced the number of repetitions (static stretching: 20.8%; ballistic stretching: 17.8%) compared with a no-stretching condition. However, Beedle et al. (2008) found no differences in maximum strength in the 1RM test of the bench press and leg press exercises in trained men when preceded by static and dynamic stretching.

Some physiological mechanisms could be responsible for explaining such reductions in strength performance after an intervention with different stretching methods including: a decrease in neural activation caused by the Golgi tendon reflex, changes in viscoelastic properties of muscle-tendon units, and the arrangement of muscle fibers (Fowles et al., 2000; Lieber, 2010). Few studies have examined the effects of different stretching methods on maximal repetition performance in an entire training session of resistance training (Sá et al., 2013). Thus, this study aimed to clarify the acute effects of passive static stretching and ballistic stretching on performance during a resistance training session. Therefore, the purpose of the present study was to compare the acute effects of passive static stretching, ballistic stretching, and of a specific warm-up, on the number of repetitions during a lower body resistance training session.

## Material and Methods

### Participants

Nine men (age: 24.3 ± 3.0 yrs, body mass: 88.8 ± 11.2 kg, body height: 189.0 ± 9.1 cm, BMI: 24.7 ± 1.4 kg/m^2^) volunteered to participate in the study. The subjects were physically active, but none had participated in regular resistance training for a minimum of six months prior to the start of the study. All of the participants answered the International Physical Activity Questionnaire (IPAQ) to determine their physical activity level. To be included in the experiment, volunteers had to meet the following criteria: not perform any type of regular physical activity other than the prescribed resistance training over the course of the study; and not consume any ergogenic aid that could influence the collection or interpretation of data. Study details were explained verbally and in writing, and all participants read and signed an informed consent form before participation in the study in accordance with the declaration of Helsinki. The study protocol was approved by the Research Ethics Committee of the Federal University of Rio de Janeiro (protocol number 101/2011).

### Procedures

The study consisted of six visits with a 48-hour rest period between following sessions. The first visit included a familiarization session including different stretching methods, a specific warm-up, and exercises that composed the resistance training session (RTS). On the second and third day, a test and retest of 1RM were performed. From the fourth visit forward, subjects were randomized and counterbalanced into three experimental protocols: a) ballistic stretching (BS) + RTS; b) passive static stretching (PSS) + RTS; and c) a specific warm-up (SW) with 20 repetitions at 30% of the 12RM load + RTS. Thirty seconds after completing the stretching protocols and a specific warm-up, the subjects started the RTS. The lower body RTS included three sets with the load adjusted to 12RM on the following exercises: leg press 45 (LP), leg extension (LE), leg curl (LC), and plantar flexion (PF). All protocols utilized a 90-second passive rest period between exercises and sets.

### Twelve Repetitions Maximum (12RM) Testing

The 12RM test protocol was performed as follows: a) a 12-repetition warm-up at 40–60% of the maximal perceived 12RM load; b) after a minute of rest period, each subject performed five repetitions at 60–80% of the maximal perceived 12RM load (Gomes et al., 2011); c) after another minute of rest, the load test was started, with each individual performing a maximum of three attempts for each exercise with a five-minute rest interval between each attempt; and d) when the subject could no longer perform the movement at the amplitude marked by the fleximeter (FLEXIMETER^®^ – Paraná – Brasil), the test was interrupted and the last complete execution prior to concentric muscular failure was considered the maximal load for the 12 repetitions. After obtaining the load for the first exercise, a 10-minute rest interval was taken before proceeding to the next exercise. Forty-eight hours after the first session, the retest was applied to assess the reproducibility of the maximal load (12RM). The execution order of the test exercises was set by the researcher for every subject and maintained throughout the entire experimental procedure. To reduce the margin of error of the 12RM test, the following strategies were adopted: a) familiarization before the test, allowing the subject to get acquainted with the data collection routine; b) instructions regarding the exercise performance techniques; c) evaluator attention to the position adopted by the subject; d) verbal encouragement; and e) measurement of the weights on a precision scale. The 12RM load was defined as the highest load achieved on both days of the test with differences smaller than 5%. In case of a greater difference, the subject returned to perform a retest again. For this purpose, all exercises utilized a movement amplitude limiter to determine the start and end positions of each exercise.

### Stretching Protocols

For each method of stretching (passive static and ballistic), three sets were performed for each muscle group (i.e., knee flexors and extensors, hip adductors, and plantar flexors). Thus, for ballistic stretching, pendulum movement’s hip adduction and abduction, hip flexion and extension, knee flexion and extension, and plantar flexion and extension were performed. Each set of ballistic stretching lasted 1 min and each pendulum motion of extension and flexion or adduction and abduction was performed in time of one second (Bacurau et al., 2009) controlled by a metronome. Because these are combined movements, each muscle group executing these pendulum movements was lengthened by 30 s in each set. For both methods of stretching, exercises were performed unilaterally on both legs with 30 s of rest. For the passive static stretching protocol, the movement was taken to a position of slight discomfort indicated by the subject and held for 30 s. It is important to note that each muscle group was stretched the same amount of time (sets and sustained time) with a total time of 90 s per muscle group (ACSM, 2011; Wallmann et al., 2012).

### Statistical Analyses

Data normality was tested by the Shapiro-Wilk homoscedasticity test (Bartlett criterion). All variables showed normal distribution and homoscedasticity. To test the load reproducibility between the 12RM test and retest, intraclass correlation coefficient was utilized (LP, r = 0.99; LE, r = 0.98; LC, r = 1.00; PF, r = 0.99). One-way ANOVAs were used to compare the effects of different experimental protocols (PSS × BS × SW) on the three-set maximal repetition numbers for each exercise and on the total RM for the resistance training session (LP, LE, LC, and PF). In case of a significant F, a Tukey HSD post hoc was performed. The statistical procedures were performed with the SPSS 20.0 program (SPSS Inc., USA) and were based on a significance level of p < 0.05.

## Results

### Total number of repetitions in each exercise

**Figure 1:**
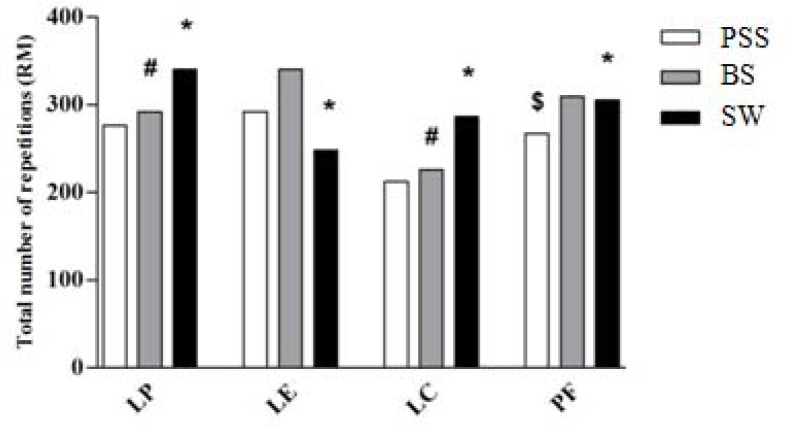
Total number of repetitions in each exercise. LP – leg press; LE – leg extension; LC – leg curl; PF – plantar flexion; PSS – passive static stretching; BS - ballistic stretching; SW – specific warm-up; ^*^ Significant difference between the SW and PSS; ^#^ Significant difference between the SW and BS; ^$^Significant difference between PSS and BS

There was a significant difference among all experimental conditions (p < 0.05). A higher number of repetitions for the SW was observed when compared to the PSS for the leg press (340 *vs*. 276; p = 0.001), leg curl (286 *vs*. 212; p = 0.001), and plantar flexion (286 *vs*. 212; p = 0.001) exercises (Figure 1). In addition, in the comparison between stretching PSS *vs*. BS, a significant reduction was observed only for the plantar flexion exercise (267 *vs*. 309; p = 0.019). Furthermore, significant increases were found between stretching BS *vs*. SW for leg press (292 *vs*. 340; p = 0.014) and leg curl (226 *vs*. 286; p = 0.002), respectively.

### Total number of repetitions in each resistance training session

**Figure 2:**
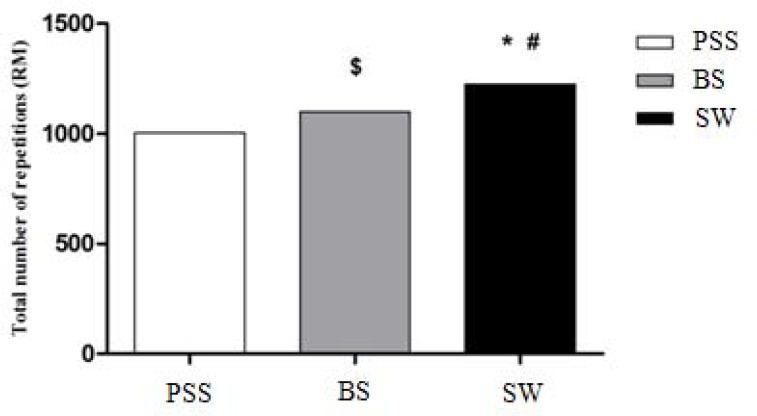
Total number of repetitions in each resistance training session. PSS – passive static stretching; BS - ballistic stretching; SW – specific warm-up; ^*^ Significant difference between PSS and BS; ^#^ Significant difference between PSS and the SW; ^$^Significant difference between BS and the SW

A higher number of repetitions was found for the SW when compared to PSS (1226 *vs*. 1003, respectively; p = 0.001). In addition, BS showed a better performance compared to PSS (1101 *vs*. 1003, respectively; p = 0.008), while the SW protocol resulted in a higher number of repetitions than BS (1226 *vs*. 1101, respectively; p = 0.002) (Figure 2).

## Discussion

The purpose of the present study was to compare the acute effects of static stretching, ballistic stretching, and a specific warm-up on the number of repetitions (12RM) in a lower body resistance training session. Accordingly, the results of the present study demonstrated that PSS caused the lowest repetition performance when compared with the BS and SW methods for all exercises in the training session. Likewise, for the sum of all repetitions in all exercises during a single training session, the lowest performance was also found in the PSS condition compared to the BS and SW. Similar results were found by Barroso et al. (2012). These authors compared the acute effects of static, ballistic, and PNF stretching on maximal strength, total volume, and maximum number of repetitions in the leg-press exercise. In terms of maximum repetitions, significant differences were found for the static stretching and ballistic stretching, with both showing a lower performance compared to the control condition (20.8% and 17.7%, respectively). These results support those of the current study because significant differences were also found between static stretching and the specific warm-up (18.8%), and between ballistic stretching and the specific warm-up (13.5%). As observed, both studies have similarities regarding stretching techniques. However, Barroso et al. (2012) used two exercises (one arm exercise and one leg exercise) whereas the present study utilized a resistance training session with four exercises for the lower body, prioritizing only one body segment. Hence, similar findings reaffirm the theory that stretching would be the main factor responsible for the decrease in performance.

Gomes et al. (2011) investigated the effects of static and PNF stretching on the maximum number of repetitions at intensities of 40, 60, and 80% of 1RM in the leg extension exercise. There were no significant differences for any of the intensities (40, 60, and 80%) when comparing static stretching with the no-stretching condition. However, in the present study, significant differences were found with the passive static stretching presenting a 15.9% higher repetition number when compared with the specific warm-up for the leg extension exercise. For static stretching, both studies used similar techniques, but as the knee extension exercise for this study was the second to be performed during the resistance training session, perhaps the muscular fatigue added to neural and mechanical effects provided by static stretching may have resulted in adverse effects on strength performance.

To examine the effects of static stretching on muscle endurance performance of the leg curl exercise, Nelson et al. (2005) conducted two experiments. In the first experiment, the authors used 40 and 60% of body weight and analyzed the maximal number of repetitions performed with and without static stretching. In the second experiment, the maximal number of repetitions was also studied with the same conditions (with static stretching and without stretching) but using 50% of body weight. For the 40% of body weight protocol, the maximal number of repetitions was 9.8% lower with stretching when compared with the no stretching condition. For 60% of body weight, the maximal number of repetitions after static stretching was 24% lower than the no stretching condition. In the second experiment using 50% of body weight, the static stretching group’s performance was 28% lower than the no stretching condition (Nelson et al., 2005). In the present study, significant differences were found among the condition with a specific warm-up having a 25.8% and 20.9% higher number of repetitions than passive static and ballistic stretching, respectively. Therefore, the effects were similar to the results of this study, which leads us to believe that the negative effects of passive static stretching on muscle endurance performance seem to be potentiated when applied to a higher overload.

For the plantar flexion exercise, significant reductions were found with passive static stretching (13.59%) when compared to ballistic stretching, and to the specific warm-up (12.45%) when compared with passive static stretching. In their study, Fowles et al. (2000) observed that after one hour the strength levels were still reduced by 9.8%. A plantar flexion exercise was the last to be performed in the present study and the entire resistance training session phase lasted approximately 50 min, thus the static and ballistic stretching effects could still have been present because the percentages found in both studies were similar.

Considering the results of all exercises that comprised the resistance training session, at least one of the conditions (passive static stretching and ballistic stretching) resulted in significant reductions when compared with the specific warm-up. These results lead us to believe that both methods affected the resistance training session performance. Thus, according to the literature, two theories would justify the results in this study and could influence the strength performance negatively as well as act at different times during the training session. For the first two exercises (leg press and leg extension), it is believed that the deficit in strength would be caused by neural responses, once we compare percentages of a decrease in performance of this study with those of Fowles et al. (2000), the results are quite similar. In order to assess the duration of the negative effects of passive static stretching on strength performance, Fowles et al. (2000) had subjects perform thirteen stretching sets, with a duration of 135 s each. Their results showed that the maximum voluntary contraction presented different performance levels for different time periods assessed. Therefore, decreases in performance of 28% immediately after stretching, 21% after 5 min, 13% after 15 min, 12% after 13 min, 10% after 45 min, and 9% after 60 min were reported. In fact, the findings of the present study are very similar to those reported by Fowles et al. (2000). These authors observed an increased performance in the number of repetitions when comparing passive static stretching *vs*. a specific warm up (18.82%), ballistic stretching *vs*. a specific warm up (13.5%) for the leg press exercise. Passive static stretching compared with the specific warm up showed a 15.92% increase in performance in the number of repetitions for the knee extension exercises. If we consider the execution time and the rest interval between sets, we notice that at the end of the second exercise (knee extension) around 20 min of training have elapsed, therefore justifying the percentages found in both studies. This performance decrease may be related to a reduction in neuronal activation induced by the Golgi tendon organ (GTO) and because of its location at the musculotendinous junction, it would be responsible for detecting the tension experienced by the muscle (Fowles et al., 2000).

In the third exercise (knee flexion), the decrease in performance could be related to the structure of muscle architecture, which may have been altered by stretching and by the mechanical stress of the resistance training session. This could promote changes in muscle fibers and biomechanical characteristics (Lieber, 2010; Hindle et al., 2012), generating physiological changes in muscle structure and consequently reducing the performance of RM in a resistance training session (Abellaneda et al., 2009; Kato et al., 2010). According to Lieber (2010), changes in mechanical structures can also lead to a decrease in performance. Therefore, when a muscle group, along with the tendons, is under the influence of a large tension for a long period of time, the viscoelastic structures are altered, causing changes in stiffness and changing the tendon structure, thereby providing variations in force transmittal (Lieber, 2010).

For the fourth and last exercise (plantar flexion and extension), it was observed that the results were again similar to those of Fowles et al. (2000). Because these muscles were not used in the other three exercises that preceded them, the gastrocnemius muscle suffered only the negative effects of stretching. Thus, the time elapsed between stretching and its execution became long, causing less of a negative effect on strength performance. As previously stated, Fowles et al. (2000) found that strength levels decreased in a similar study (decrease of 13.59% for passive static stretching *vs*. ballistic stretching, and a decrease of 12.45% in performance for ballistic stretching compared to a specific warm-up). Thus, it is believed that the same effects caused by a reduction in neural activation induced by the GTO are present in this situation, but to a lesser degree because of the longer time elapsed between the intervention with stretching and performing the exercise. With regard to the muscle architecture, because it was perhaps not used as much as the other muscles, the gastrocnemius muscle was not exposed to high loads or tensions. These conditions might not have caused the viscoelastic structure changes to the point of causing an alteration in the stiffness of the tendon. For the three sets of four exercises, significantly lower numbers of RM were found for passive stretching and ballistic stretching compared to the specific warm-up (11.31% and 9.75% respectively), and significantly lower for passive stretching compared to ballistic stretching (1.72%).

The method that showed the least decrease in force development was the specific warm-up, whereas passive static stretching showed to be most inefficient for repetition performance. The explanation for this negative performance may be due to the presence of neural fatigue (due to the stretching method) in addition to muscular fatigue (due to the RTS). The main limitation of this study was that the short rest interval between sets and exercises could affect the RTS adaptations as well as muscle architecture, therefore the ultrasound method (pennation angle, fascicle length, or muscle size) was not examined. Nevertheless, these results may assist health professionals who prescribe resistance training and those who work in rehabilitation to take precaution regarding the effects of stretching exercises performed before resistance training. The results are of greater significance when training is directly dependent on strength performance.

## Conclusions

In conclusion, static and ballistic stretching should not be recommended before a resistance training session because according to the presented results, a pre-exercise stretching session hinders the subsequent resistance training performance. Further research is needed to investigate the influence of different stretching strategies on an upper body resistance training session, in different populations, and of different training levels.

## Figures and Tables

**Table 1:** Stretching protocols in static and ballistic methods

	Static Stretching	Ballistic Stretching
Knee extensors	The subject was placed in a prone position on a padded table and performed unilateral knee flexion combined with a hip extension.	Standing, the subjects performed unilateral pendular movements of hip and knee flexion followed by the hip and knee. The sets lasted for 1 min and each pendulum movement was performed in 1 s controlled by a metronome.
Knee flexors	The subject was placed in a supine position on a padded table and performed unilateral hip flexion with an extended knee.	Standing, the subjects performed pendular movements flexion and extension of the unilateral hip with an extended knee. The sets lasted for 1 min and each pendulum movement was performed in 1 s controlled by a metronome.
Hip adduction	The subject was placed in a supine position on a padded table and performed a unilateral lateral hip rotation combined with a hip flexion with a flexed knee.	Standing, the subjects performed unilateral pendular movements of hip adduction and abduction. The sets lasted for 1 min and each pendulum movement was performed in 1 s controlled by a metronome.
Plantar Flexion	The subject was placed in a supine position on a padded table and performed a unilateral plantar flexion with an extended knee.	The subject was placed supine on an exercise mat, with one knee bent supported on a step (30 cm). Then, the subject performed a movement of plantar flexion and extension for 1 min. Each movement of plantar flexion or extension lasted for 1 s, and was controlled by a metronome.

## References

[b1-jhk-45-177] Abellaneda S, Guissard N, Duchateau J (2009). The relative lengthening of the myotendinous structures in the medial gastrocnemius during passive stretching differs among individuals. J Appl Physiol.

[b2-jhk-45-177] American College of Sports Medicine. ACSM ś position stand (2011). Quantity and quality of exercise for developing and maintaining cardiorespiratory, musculoskeletal, and neuromotor fitness in apparently healthy adults: guidance for prescribing exercise. Med Sci Sports Exerc.

[b3-jhk-45-177] Ayala F, Ste CM, Sainz BP, Santonja F (2013). Acute effects of static and dynamic stretching on hamstring eccentric isokinetic strength and unilateral hamstring to quadriceps strength ratios. J Sports Sci.

[b4-jhk-45-177] Bacurau RFP, Monteiro GA, Ugrinowitsch C, Tricoli V, Cabral LF, Aoki MS (2009). Acute effect of a ballistic and a static stretching exercise bout on flexibility and maximal strength. J Strength Cond Res.

[b5-jhk-45-177] Barroso R, Tricoli V, Dos Santos Gil S, Ugrinowitsch C, Roschel H (2012). Maximal strength, number of repetitions, and total volume are differently affected by static-, ballistic-, and proprioceptive neuromuscular facilitation stretching. J Strength Cond Res.

[b6-jhk-45-177] Beedle B, Rytter SJ, Healy RC, Ward TR (2008). Pretesting static and dynamic stretching does not affect maximal strength. J Strength Cond Res.

[b7-jhk-45-177] Chen CH, Chen TC, Jan MH, Lin JJ (2014). Acute Effects of Static Active or Dynamic Active Stretching on Eccentric Exercise-Induced Hamstring Muscle Damage. Int J Sports Physiol Perform.

[b8-jhk-45-177] Costa PB, Herda TJ, Herda AA, Cramer JT (2014). Effects of dynamic stretching on strength, muscle imbalance, and muscle activation. Med Sci Sports Exerc.

[b9-jhk-45-177] Cramer JT, Beck TW, Housh TJ, Massey LL, Marek SM, Danglemeier S, Purkayasthac S, Culbertsond JY, Fitzc KA, Egana AD (2007). Acute effects of static stretching on characteristics of the isokinetic angle–torque relationship, surface electromyography, and mechanomyography. J Sports Sci.

[b10-jhk-45-177] Cramer JT, Housh TJ, Coburn JW, Beck TW, Johnson GO (2006). Acute effects of static stretching on maximal eccentric torque production in women. J Strength Cond Res.

[b11-jhk-45-177] Cramer JT, Housh TJ, Johnson GO, Miller JM, Coburn JW, Beck TW (2004). Acute effects of static stretching on peak torque in women. J Strength Cond Res.

[b12-jhk-45-177] Fowles J, Sale D, MacDougall J (2000). Reduced strength after passive stretch of the human plantar flexors. J Appl Physiol.

[b13-jhk-45-177] Franco BL, Signorelli GR, Trajano GS, Oliveira CG (2008). Acute effects of different stretching exercises on muscular endurance. J Strength Cond Res.

[b14-jhk-45-177] Gomes TM, Simão R, Marques MC, Costa PB, Novaes JS (2011). Acute effects of two different stretching methods on local muscular endurance performance. J Strength Cond Res.

[b15-jhk-45-177] Hindle KB, Whitcomb TJ, Briggs WO, Hong J (2012). Proprioceptive neuromuscular facilitation (PNF): Its Mechanisms and effects on range of motion a and muscular function. J Hum Kinet.

[b16-jhk-45-177] Kato E, Vieillevoye S, Balestra C, Guissard N, Duchateau J (2011). Acute effect of muscle stretching on the steadiness of sustained submaximal contractions of the plantar flexor muscles. J Appl Physiol.

[b17-jhk-45-177] Lieber RL (2010). The physiological basis of rehabilitation: skeletal muscle structure, function, & plasticity.

[b18-jhk-45-177] Manoel ME, Harris-Love MO, Danoff JV, Miller TA (2008). Acute effects of static, dynamic, and proprioceptive neuromuscular facilitation stretching on muscle power in women. J Strength Cond Res.

[b19-jhk-45-177] Nelson AG, Kokkonen J, Arnall DA (2005). Acute muscle stretching inhibits muscle strength endurance performance. J Strength Cond Res.

[b20-jhk-45-177] Ogura Y, Miyahara Y, Naito H, Katamoto S, Aoki J (2007). Duration of static stretching influences muscle force production in hamstring muscles. J Strength Cond Res.

[b21-jhk-45-177] Rhea MR (2004). Determining the magnitude of treatment effects in strength training research through the use of the effect size. J Strength Cond Res.

[b22-jhk-45-177] Rubini EC, André LLC, Paulo SCG (2007). Effects of stretching on strength performance. Sports Med.

[b23-jhk-45-177] Sá MA, Gomes TM, Bentes CM, Costa e Silva G, Rodrigues Neto G, Novaes JS (2013). Acute effect of static and proprioceptive neuromuscular facilitation stretching methods in the maximum number of repetitions in a single strength training session performance. Motricidade.

[b24-jhk-45-177] Sekir U, Arabaci R, Akova B, Kadagan SM (2010). Acute effects of static and dynamic stretching on leg flexor and extensor isokinetic strength in elite women athletes. Scand J Med Sci Sports.

[b25-jhk-45-177] Wallmann HW, Christensen SD, Perry C, Hoover DL (2012). The acute effects of various types of stretching static, dynamic, ballistic, and no stretch of the iliopsoas on 40-yard sprint times in recreational runners. Int J Sports Phys Ther.

[b26-jhk-45-177] Winchester JB, Nelson AG, Kokkonen J (2009). A single 30-s stretch is sufficient to inhibit maximal voluntary strength. Res Q Exerc Sport.

[b27-jhk-45-177] Yamaguchi T, Ishii K, Yamanaka M, Yasuda K (2006). Acute effect of static stretching on power output during concentric dynamic constant external resistance leg extension. J Strength Cond Res.

